# When beggars are choosers—How nesting of a solitary bee is affected by temporal dynamics of pollen plants in the landscape

**DOI:** 10.1002/ece3.4116

**Published:** 2018-05-15

**Authors:** Anna S. Persson, Florence Mazier, Henrik G. Smith

**Affiliations:** ^1^ Centre of Environmental and Climate Research Lund University Lund Sweden; ^2^ Department of Environmental Geography Jean Jaurès University Toulouse France; ^3^ Department of Biology Lund University Lund Sweden

**Keywords:** agri‐environment schemes, central‐place forager, farmland biodiversity, organic farming, pollen foraging, pollinator, red mason bee

## Abstract

Wild bees are declining in intensively farmed regions worldwide, threatening pollination services to flowering crops and wild plants. To halt bee declines, it is essential that conservation actions are based on a mechanistic understanding of how bee species utilize landscapes. We aimed at teasing apart how foraging resources in the landscape through the nesting season affected nesting and reproduction of a solitary bee in a farmland region. We investigated how availability of floral resources and potentially resource‐rich habitats surrounding nests affected nest provisioning and reproduction in the solitary polylectic bee *Osmia bicornis*. The study was performed in 18 landscape sectors dominated by agriculture, but varying in agricultural intensity in terms of proportion of organic crop fields and seminatural permanent pastures. Pasture‐rich sectors contained more oak (*Quercus robur*), which pollen analysis showed to be favored forage in early season. More oaks ≤100 m from nests led to higher proportions of oak pollen in nest provisions and increased speed of nest construction in early season, but this effect tapered off as flowering decreased. Late‐season pollen foraging was dominated by buttercup (*Ranunculus* spp.), common in various noncrop habitats. Foraging trips were longer with more oaks and increased further through the season. The opposite was found for buttercup. Oak and buttercup interacted to explain the number of offspring; buttercup had a positive effect only when the number of oaks was above the mean for the studied sectors. The results show that quality of complex and pasture‐rich landscapes for *O. bicornis* depends on preserving existing and generating new oak trees. Lignose plants are key early‐season forage resources in agricultural landscapes. Increasing habitat heterogeneity with trees and shrubs and promoting suitable late‐flowering forbs can benefit *O. bicornis* and other wild bees active in spring and early summer, something which existing agri‐environment schemes seldom target.

## 1 INTRODUCTION

Wild insects, in particular bees, are essential pollinators of crops (Klein et al., 2007) and wild plants (Ollerton, Winfree, & Tarrant, 2011). Wild bee declines have mainly been attributed to agricultural intensification causing a reduced availability and quality of foraging and nesting habitat, in combination with pesticide use (Potts et al., 2016). Because bees are central‐place foragers, they especially suffer from spatial separation of forage and nesting habitat caused by landscape simplifications through agricultural intensification (Brown & Paxton, 2009; Kremen, Williams, & Thorp, 2002). However, central‐place foraging bees may also adapt their spatial use of foraging patches to patch quality, using high‐quality resources such as ephemeral mass‐flowering crops at larger distances, compared to more scattered but continuous flower resources, in, for example, seminatural habitats (cf. Olsson & Bolin, 2014; Olsson, Bolin, Smith, & Lonsdorf, 2015). In addition to spatial variation in forage, bees will also be affected by temporal variation in forage throughout their activity and nesting season. Landscape simplification may result in a higher spatial autocorrelation of land use and as a result increased temporal variation in flower resources, with negative impacts on bees (Mallinger, Gibbs, & Gratton, 2016). Thus, resource use by bees in changing landscapes is complex and needs to be elucidated by a mechanistic understanding of how spatial and temporal variation in flower resources affect bee foraging and, as a consequence, their fitness and population dynamics. Such knowledge may in turn be used to inform the design of measures to mitigate bee declines (Wood, Holland, & Goulson, 2015, 2016).

A major aim of agri‐environment schemes (AES) is to preserve farmland biodiversity (Batáry, Dicks, Kleijn, & Sutherland, 2015). Organic farming, a form AES, may benefit wild bees through a decreased in‐field farming intensity leading to higher abundance and diversity of flowering herbs in and adjacent to fields throughout landscapes (Kennedy et al., 2013; Rundlöf, Edlund, & Smith, 2010). Other AES may benefit bees by increasing landscape complexity through the preservation, restoration, and management of seminatural habitats containing flowers and nesting resources, such as permanent grasslands and noncrop field borders (Kennedy et al., 2013; Persson & Smith, 2013). As bees also use massively abundant entomophilous crops such as oilseed rape (OSR), variation in the amount of such crops may obscure any effect of organic farming or seminatural habitat (cf. Holzschuh, Dormann, Tscharntke, & Steffan‐Dewenter, 2013), but possibly only during part of the season (Jauker, Peter, Wolters, & Diekötter, 2012) and for some pollinator species (Riedinger, Mitesser, Hovestadt, Steffan‐Dewenter, & Holzschuh, 2015).

Solitary bees may be particularly affected by landscape intensification (Le Féon et al., 2010) because they experience landscapes at smaller spatial scales compared to social bees (Gathmann & Tscharntke, 2002; Zurbuchen, Landert et al., 2010) and often construct nests, lay eggs, and forage to provision for these during a period of several weeks (Linowski, Cederberg, & Nilsson, 2004). The habitat quality experienced by the egg‐laying females will vary over their flight season (Mandelik, Winfree, Neeson, & Kremen, 2012; Williams & Tepedino, 2003), both as a direct result of plant flowering phenology and indirectly from management intensity and practices affecting temporal patterns of flower abundance (Williams & Kremen, 2007). Compared to monolectic or oligolectic species (i.e., species foraging from only a single plant species or from only a few plant species or genera, respectively), polylectic species using many plant species may be better adapted to such seasonal variation, because they can utilize pollen and nectar from several plant families and thereby compensate for a loss, or lack, of favored forage (Linowski et al., 2004; Williams & Kremen, 2007). However, the life history of a species may limit the degree to which such compensation can buffer fitness consequences. For example, several solitary bee species (e.g., *Osmia* spp.) have protandrous emergence (males emerging before females). Such species lay female eggs at the back of nests and predominantly early in the season and then male eggs later and toward the nest opening (Giejdasz, Fliszkiewicz, Bednářová, & Krishnan, 2016; Torchio & Tepedino, 1980). Female bees are larger than males and require more pollen per egg for development (Radmacher & Strohm, 2010). This sequential investment in female vs. male offspring may, in accordance with sex allocation theory (Rosenheim, Nonacs, & Mangel, 1996), be an adaptation to resource plant phenology, pollen nutritional value, and declining foraging ability of aging females to provision for larger female offspring (O'Neill, Delphia, & Pitts‐Singer, 2015; Roulston & Cane, 2000; Torchio & Tepedino, 1980). Therefore, high availability of preferred pollen close to the nest early in the nesting season is expected to be crucial to maintain production and fitness of daughters. Resources later in the season may be more readily interchangeable as male fitness does not rely on body size (Seidelmann, 2014). Such seasonal variation in resource requirements complicates evaluation of general habitat or landscape quality. It is therefore important to investigate how the consequences of resource availability and agricultural management impact bee foraging throughout the nesting season.

We aimed at investigating how availability of flower resources, and of habitats assumed to provide flower resources, affected foraging, reproductive success, and population size of a solitary bee, the polylectic red mason bee *Osmia bicornis,* throughout its nesting season. To do so, we identified forage plant species in pollen provisions from *O. bicornis* nests (i.e., pollen provided by the female bee to cater for offspring development) and measured foraging trip times, nest‐building speed, reproductive output, and population size of *O. bicornis* nesting in trap nests in permanent field borders in 18 different landscape sectors (500 m radius). We contrasted three landscape types: predominantly conventionally managed landscapes with a low proportion of seminatural permanent pastures, with similar landscapes that either were predominantly organically managed or contained a high proportion of seminatural permanent pastures. We assumed that both organic farming and more seminatural pastures resulted in more flowering plants suitable as forage at a shorter distance from nests. We also directly measured the local (≤100 m of the nest) abundance of plants providing pollen forage, which we expected to be a mix of trees, shrubs, and forbs. We determined the amount of mass‐flowering crops (autumn‐sown OSR) at both spatial scales. We investigated whether bee population, fitness, and foraging variables were related to the habitat availability measured at the wider landscape scale and/or to locally measured availability of plants for pollen foraging. We expected a higher number of nesting females, with higher reproductive success, more female‐skewed sex ratio in offspring, and more efficient foraging and nest construction when local flower resources were more abundant and/or in landscapes characterized by organic farming, or permanent pastures, and that OSR would produce similar results. We expected that the plant species used would change over the season and that responses to plant abundances would therefore vary depending on the flowering season of pollen plants.

## 2 METHODS

### 2.1 Study organism

**Figure 1:**
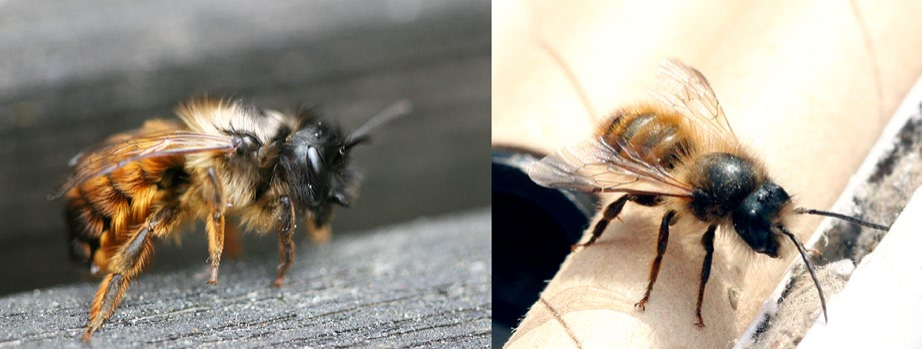
The study organism, *Osmia bicornis*, the red mason bee. Left panel: a newly hatched female, right panel: a newly hatched male on top of cardboard nesting straws. Photograph: Anna S. Persson

The red mason bee *O. bicornis* (previously *O. rufa,* Figure 1) is a solitary, polylectic bee common throughout Central and Northern Europe, with an annual life cycle and a nesting period from mid‐April throughout June depending on the region (Linowski et al., 2004; Radmacher & Strohm, 2010; Raw, 1974). After mating, each female constructs a nest, for example, in a hollow twig, tubular insect burrow, or crevice in mortar (Linowski et al., 2004; Raw, 1972). Eggs are laid in sequence, in separate brood cells together with a provision of pollen and sealed with loam, to form a gallery of 10–20 cells. A female may construct several nests, and the proportion of female eggs is higher in nests constructed at the beginning of the nesting period, compared to nests constructed later (Giejdasz et al., 2016). Females (larvae, pupae, and adults) are larger than males and require more pollen for development (Radmacher & Strohm, 2010; Seidelmann, 2014). The offspring develops into adults before the end of summer and hibernates inside the nest to emerge the following spring (Radmacher & Strohm, 2010). Preferred pollen plants are species of *Quercus* (oak), *Ranunculus* (e.g., buttercup), *Acer* (maple), and *Aesculus hippcastanum* (horse chestnut), while species of Brassicaceae and Rosaceae are main sources of nectar and potential secondary pollen sources (Jauker et al., 2012; Radmacher & Strohm, 2010; Raw, 1974). *O. bicornis* has also been reported to visit, for example, *Lamium album*,* Papaver dubium*,* Salix* spp., *Syringa vulgaris, Taraxacum*, and *Trifolium repens* (Pettersson, Cederberg, & Nilsson, 2004; Radmacher & Strohm, 2010; Raw, 1974), and to benefit from oilseed rape (Holzschuh et al., 2013; Jauker et al., 2012).

### 2.2 Study sites

**Figure 2:**
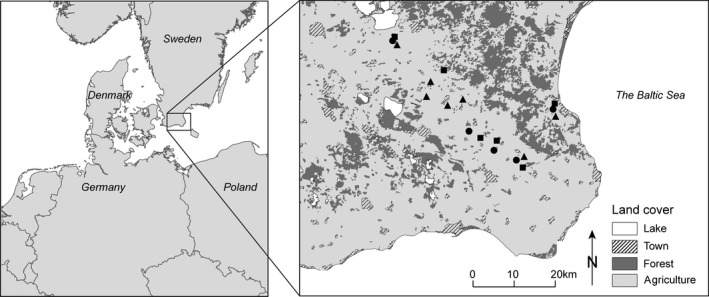
The study area in the southeastern part of the province of Scania. The seven organic sectors (triangles), five conventional sectors (squares), and six pasture‐rich sectors (circles) were well interspersed and situated in landscapes dominated by farmland (light gray), with little forest (dark gray) or urban areas (diagonal lines)

The study was carried out in the province of Scania, southernmost Sweden (Figure 2), a region largely dominated by agriculture, but with a large variation in both land‐use intensity and landscape complexity (Persson et al., 2010). Landscape sectors (hereafter referred to as sectors) were situated within an area with relatively high landscape complexity, containing small farms with mixed farming. Land use consisted of a mix of annual crop fields, grass–clover leys for silage production, permanent pastures, some small woodlots, and linear seminatural elements (e.g., permanent field borders and road verges).

Previous studies indicate that resource availability at distances up to 500 m likely affects fitness in solitary bees in general, including *Osmia* spp. (Gathmann & Tscharntke, 2002; Williams & Kremen, 2007; Zurbuchen, Landert et al., 2010). We therefore defined landscape types based on land use within a radius of 500 m, with centers of sectors separated by >1,000 m. Land‐use data were obtained from the Integrated Administration and Control System (IACS, a yearly updated database on spatial extent and land use of all registered farmland fields in Sweden from the Swedish Board of Agriculture) and processed in ArcGIS 9.3 (ESRI) and MATLAB (The MathWorks Inc.). Based on the 2007 IACS, we selected six sectors each of three different landscape types: conventional, organic, and pasture‐rich. One farm within a conventional sector converted to organic management in 2007 (registered in IACS 2008); we therefore considered that sector organic, resulting in five conventional, seven organic, and six pasture‐rich sectors. All three landscape types were well interspersed spatially (Figure 2). Based on 2008 IACS, we could see that as intended, (1) both conventional and organic sectors were dominated by arable fields and had very little seminatural pasture, (2) almost 50% of arable fields in organic sectors were under organic management, and (3) the pasture‐rich sectors were much richer in seminatural pasture than both conventional and organic sectors (Table 1). To evaluate how well the 500‐m scale described the sectors, we extracted data on land use at 100 and 1,000 m and checked for correlations between the three spatial scales. We also extracted data on the area of OSR. Results showed that land‐use variables at these spatial scales were strongly correlated (Appendix S1). Data at the 500‐m scale therefore well represent the landscape.

**Table 1:** Land cover during 2008 used to categorize landscape sectors (500 m radius), showing the proportion arable fields (including annual crops, leys, and fallow (one field only)), organic arable fields (annual crops and leys), or permanent grazed pasture

Landscape type	Arable fields	Organic arable fields	Permanent pasture
Conventional (*N* = 5)	0.84 ± 0.073	0 ± 0	0.023 ± 0.030
Organic (*N* = 7)	0.82 ± 0.056	0.51 ± 0.084	0.055 ± 0.044
Pasture (*N* = 6)	0.39 ± 0.067	0.017 ± 0.042	0.43 ± 0.11

Data were obtained from the Integrated Administration and Control System (IACS, Swedish Board of Agriculture). Mean values and standard deviations are shown.

### 2.3 Study setup

To increase the likelihood of nest establishment and control for nest substrate, we used so‐called trap nests (Oxford Bee Company^™^) seeded with *O. bicornis* pupae (cf Williams & Kremen, 2007) collected the previous year (2007) in the same region and overwintered in an open shed in ambient temperature. Trap nests consisted of plastic tubes, Ø 7.0 cm, length 16.8 cm and filled with ca 30–35 cardboard straws, Ø 0.6–0.9 cm, length 15.3 cm. Straws were lined with a thin white paper that could be pulled out and investigated without destroying brood cells. Two trap nests were attached to a 1.5‐m wooden pole, and four such poles were placed in the center of each of the 18 sectors, ca 50–150 m apart depending on where suitable habitat was found. We considered suitable habitat to be permanent noncrop borders of fields and pastures containing some low trees and shrubs, rendering protection from agricultural management and some shelter from wind. All trap nests were positioned toward the southeast. The adjacent land use depended on landscape type; that is, nests were placed in borders to conventional fields in conventional sectors, organic fields in organic sectors, and pasture in pasture‐rich sectors. Each of the four sets of nests was seeded with two female and two male pupae, that is, in total eight females and eight males per sector. We monitored the nests every second day until pupae had hatched and we could confirm nest establishment. Each cardboard straw with an actively nest‐building female was given a number, noted with a color code on the brim of the straw, to allow identification during filming.

### 2.4 Nesting, foraging, and reproductive data

In the field, we collected data on nest‐building speed, pollen foraging trip times, and the number of nest‐building females. Each nest was visited every third day except when it rained a full day, in which case the visit was postponed until the next day. The time of day of visits (mornings or afternoons) was rotated between sectors, and we noted the exact time of each visit to each trap nest.

Measurements of nest‐building were acquired by pulling out the white paper lining from cardboard straws, marking the total length built so far, and measuring the length of the nest built since the previous visit. The volume built between measurements was calculated, and the total time between measurements was noted. At the same visit, data on foraging trip duration were obtained by filming the entrance of trap nests for ~45 min using HD cameras (JVC, GZ‐MG5775E) placed on tripods in front of the trap nest. Films were processed with the Observer XT (Noldus Information Technology) to extract data on foraging trip duration. Trips were considered to be for foraging if a female returned with pollen on her abdomen. Filming took place during the peak of *O. bicornis* activity May 11–30, while monitoring of nest‐building continued until June 17, when activity had practically stopped.

As an estimation of the number of nest‐building females per sector, we summed the maximum number of females simultaneously filmed (i.e., visible in the same film sequence but at different nests), while depositing pollen and/or loam at each nest pole. We thus assume that a female is constructing only one nest at a time. This is likely a slight underestimation of the total number of females, because there may have been females who started nesting later in the season, not overlapping with the nesting of early nesters.

To estimate the total number of offspring produced, all cardboard straws sealed with loam were brought to a field station during late July. There, we put them into a cardboard box for overwintering in an open shed (ambient temperatures). The following spring nests were brought to the laboratory and kept in an incubation room with controlled temperature until they were opened to count offspring. Because of an incident with the temperature control during a weekend, some bees hatched before we could open the nests. The sex of pupae was therefore determined either by morphology of unhatched bees (males have yellow hair in the face) or by the size of pupae of hatched bees. We checked how well this method worked by first measuring size (length and diameter) of pupae for which we could also determine sex by morphology (49 males, 38 females). The accuracy of using size of pupae for sex determination was found to be 95%. We also used the size measurements to estimate the difference in volume between female and male pupae because we wanted to be able to take into account the larger amount of resources provided for daughters. We found that female pupae were larger by a factor of 1.6. Therefore, we modeled the effects of land use and flower resources both on the number of offspring and on the number of males plus females multiplied by 1.6. Any unmarked (i.e., not previously measured or filmed) nests were allowed to hatch, and offspring were pinned and determined to species and sex. Some of these unmarked nests did contain *O. bicornis* and these were included in data on the total number of offspring.

In one pasture‐rich sector, rooks pulled out some cardboard straws from the nest tubes on a few occasions. We therefore removed this sector from analyses of reproduction and nest‐building speed (*N* = 4 observations) but included data on foraging trip times from this sector because there was undisturbed activity recorded during filming. To remove outliers of observations of nest‐building, we excluded built nest volumes per three days of 0 cm^3^ (*N* = 6) or >21 cm^3^ (*N* = 1) as these observations likely were based on nests where activity had stopped or where we had missed the initiation of nest‐building. To remove outliers of foraging trip times, we excluded observations of <15 (*N* = 15) or >1,800 s (*N* = 6). The short trips may have originated from bees being disturbed when entering the nest and therefore making a new entry with the same pollen load. The very long trips were removed because they appeared as outliers on a normal plot, with both untransformed and transformed (log, root) data.

To be able to control for effects of temperature on foraging trip times, we measured local temperature with a portable weather station (Oregon Scientific, BAR688HG) during filming of foraging trips. To control for weather effects on nest‐building speed, we obtained temperature data from an SMHI (Swedish Meteorological and Hydrological Institute) weather station in the study region. The mean maximum temperature and the mean amount of rainfall for the days between measurements, that is, the period during which a certain section of the nest was built, were calculated and used in statistical models.

### 2.5 Local flower surveys

The abundance of flowers per species surrounding each nest pole was estimated at three times: May 12–14, May 20–22, and June 16–17. We surveyed the total area within a radius of 100 m around each nest pole and estimated the total number of “flower units” (i.e., flower heads, umbels, or racemes, depending on plant morphology) per species of all herbaceous flowering plants in amounts of 1–9, 10–99, 100–999, 1,000–9,999, 10,000–49,000, or >50,000. We also counted the number of flowering trees per species. The 100 m radius was chosen to reflecting the immediate foraging landscape of bees while making a quantitative survey feasible.

### 2.6 Collection and analysis of pollen from nests

**Figure 3:**
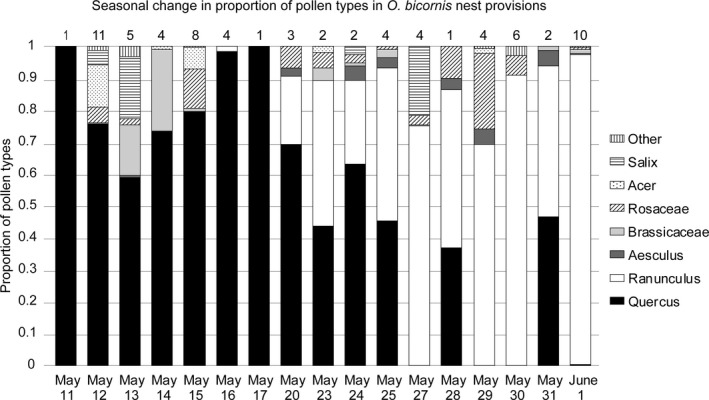
The seasonal change in pollen provisioning by *O. bicornis*. Bars show the proportional contribution of plant species or groups of plant species, to the pollen found in brood cells provisioned between May 11 and June 1, averaged per date. Numbers above bars show sample size. In total, 72 cells from the 18 landscape sectors were analyzed and 100 pollen grains were counted per sample. No samples were obtained from May 18, 19, 21, 22, or 26. Buttercup (*Ranunculus*) and oak (*Quercus*) dominate samples

To discern which plant species *O. bicornis* females used for provisioning of brood cells, we analyzed pollen taken from one brood cell each of 72 nests, representing three to five nests from each of the 18 study sectors. Samples were taken from brood cells constructed on a known date between May 11 and June 1, that is, spanning most of the study period and the period of filmed nesting activity. Samples were preserved in 70% ethanol and processed with acetolysis, and slides were prepared with glycerol as medium. Hundred pollen grains per slide were determined to species or to “pollen group” according to reference slides at the Department of Geology, Lund University, and literature (Eide, 1981; Gaillard, unpublished; Moore, Webb, & Collinson, 1991; Punt & Clark, 1984; Reille, 1992, 1995, 1998). The plant species found to dominate the samples were oak (*Quercus robur*) and buttercup (*Ranunculus* spp.) and to a lesser degree other trees (e.g., *Salix* spp. and Rosaceae) and Brassicaceae (mostly OSR, but also other species; see Section 2.1, Figure 3). Based on these results, we decided to use abundance of oak, buttercup, Brassicaceae, and flowering trees combined (except for oak), in further analyses. These are hereafter referred to as preferred pollen plants.

### 2.7 Statistical analyses

Statistical analyses were carried out in SAS 9.3 (SAS Institute Inc., Cary, NC, USA) and R (RStudio Inc., Boston, MA, USA). We analyzed whether the local abundance of preferred pollen plants affected the species of pollen found in nest provisions (arcsin square‐root‐transformed proportions) using linear mixed models (SAS Proc Mixed, normal distribution), with random factor sector id.

Data on foraging resources and land use were obtained both at 100 m (direct observations) and at 500 m (land‐use database). We evaluated whether these measures were uncorrelated and therefore could be used simultaneously in analyses or whether they were correlated and thus could not be used in the same model. To do so, we ran correlations (SAS Proc Corr) between abundance of preferred pollen plants measured at the local level (100 m), landscape type, and land use extracted at landscape scale (500 m). The abundance of oak differed between landscape types, and the abundance of OSR was highly correlated on the 500‐ and 100‐m scales. Consequently, we ran models for nesting and reproduction either on the 500‐m landscape scale with land‐use data (fixed factors: landscape type, OSR, noncrop field borders) or on the 100‐m local scale with surveyed plants (fixed factors: oak, buttercup, Brassicaceae, flowering trees).

We used the following models to analyze whether response variables related to the nesting population size and reproductive output were affected by local flower abundance and/or landscape‐scale land‐use variables: Generalized mixed models (SAS Proc Glimmix) were used to model effects on the number of nest‐building females per sector (assuming Poisson distribution), the proportion female offspring (females/individuals; assuming Binomial distribution), and the reproductive output per female (built cells; assuming Poisson distribution and offset by the (log) number of nesting females). We included sector id as an observation‐level random factor to account for overdispersion (Harrison, 2014). The measures of reproductive output summed per sector (brood cells, female offspring) were analyzed with general linear models (SAS Proc Mixed, normal distribution). Because we assume the larger females to be costlier to produce in terms of pollen collection, the reproductive output per sector was also analyzed including an adjustment for the larger volume of female pupae, that is, response variable = males + females × 1.6 (see above). Fixed effects were evaluated using F‐tests with the denominator degrees of freedom estimated with the Kenward–Roger method for all models described above.

The response variables nest‐building speed and foraging trip time were measured between two specific days and on one specific day, respectively. It was therefore necessary to model these variables at the level of an individual nest and per time period or day, to be able to control for covariates (time of day and temperature). Thus, effects of local abundance of pollen plants and land use measured at the landscape scale on foraging trip times and nest‐building were modeled using linear mixed models at the level of individual nest and day. For nest‐building, we used the volume built between two measurements as the response variable and included the time elapsed between measurements in the model to be able to account for variation in the time females were able to build nests. As brood cells can be of different size depending on the amount of pollen added, we assume volume to be closely related to the actual amount of pollen necessary to gather, and thus potentially be more affected by the abundance of pollen plants than the number of cells would be. Fixed factors were the abundance of preferred pollen plants and the variables controlling for weather. Because individual trap nests were nested within nest poles, and poles were nested within sectors, we included these as random factors. To be able to handle estimates of these random factors, which sometimes were estimated as negative, we used MCMC analysis (R MCMCglmm), with a Cauchy prior, a burn‐in of 15,000, a thinning of 5,000 and 5 × 10^6^ iterations (R MCMCglmm packages lme4 and MCMCglmm, normal distribution) in which random factors were constrained to be positive. The continuous variables of foraging resources were all measured at the nest pole level (e.g., *N* oaks within 100 m, area OSR within 500 m). With the nested design and required random structure, this results in effects assessed across poles within sectors and not across sectors. To verify the results, we therefore ran additional models on foraging trips and the nest‐building, with predictors centered on the group‐level mean, that is, the mean of the four nest poles in a sector, and also included the difference from the mean for each nest pole.

## 3 RESULTS

In three of the 18 sectors, all four nest poles contained nesting females, in 11 sectors, three poles were inhabited, and in four sectors, two poles were inhabited. In total, 218 cardboard straws containing nesting *O. bicornis* females were monitored. Of these, 164 were filmed, while females provisioned and constructed nests. Based on films and notes of the number of females simultaneously constructing nests, a minimum total of 146 individual females were observed nesting, with a mean of 8.1 ±  std 4.2 (range: 3–17) females per sector. Thus, we assume that several females constructed more than one nest. In total, 3,394 offspring were counted the following spring: 1,146 females, 1,743 males, and 505 individuals that did not pupate or fully develop into adults and could not be determined to sex. There was a mean ratio (±std) of females to total hatched individuals of 0.40 (±0.08), calculated per sector.

### 3.1 Pollen provisioning and resource availability

*Osmia bicornis* pollen foraging was highly dominated by oak early in the season (mid‐May), followed by buttercup (late May to early June; Figure 3). In fact, 27 of the 39 sampled brood cells provisioned between 11th and 23rd May contained >90% oak pollen. Oak pollen accordingly constituted a mean of 76% (std 40%) of pollen per cell in the 39 cells. Similarly, buttercup constituted >90% of pollen found in 21 of 33 cells provisioned between 24rd May and 1st June, with a mean of 75% (std 36%) of pollen per cell in the 33 cells. These results fit well in time with data from aerial surveillance of pollen, which show that oak flowering period in this region lasted between May 10 and May 30, with a likely peak around May 13–18 (Åslög Dahl, Gothenburg University, pers. commun.). Flower surveys showed that buttercup had started to flower by May 20–22 and continued to do so until mid‐June.

The proportion of oak pollen in sampled nests (arcsin square‐root‐transformed) was significantly positively related to the (log) abundance of oak trees within 100 m of nest (estimated coefficient (EC) ± *SE* = 0.15 ± 0.054; *F*_1,66.8_ = 7.66; *p *=* *.0073) and decreased over the season (effect of log date: EC = −1.22 ± 0.17; *F*_1,52.9_ = 3.35; *p *<* *.0001). In contrast, the proportion of buttercup pollen in nests was unrelated to the (log) abundance of buttercup surrounding the nest (EC *=* −0.00042 ± 0.023; *F*_1,15.4_ = 0.00, *p *=* *.99), but did increase later in the season (effect of log date: EC *= *1.50 ± 0.11; *F*_1,55.5_ = 188.14, *p *<* *.0001).

The analyses of differences between landscape types in availability of locally surveyed resources showed that the number of oak trees was higher in the pasture‐rich sectors (mean 6.13 ± std 2.98) than in the conventional (1.10 ± 2.19) or organic ones (1.93 ± 3.42) (*F*_2,15_ = 4.73, *p *=* *.026). There was no difference between the landscape types in the summed abundance of other tree species that flowered during the surveys (*F*_2,15_ < 0.01; *p *>* *.99), nor of the number of flowering buttercups (*F*_2,15_ = 1.55, *p *=* *.24), or of Brassicaceae flowers, mainly OSR (*F*_2,15_ = 2.14, *p *=* *.15). For resources measured at the landscape scale, neither the area of OSR fields nor the length of field borders (a proxy for linear seminatural habitats) differed between landscape types (*F*_2,15_ = 2.50, *p *=* *.12 and *F*_2,15_ = 1.09, *p *=* *.36, respectively).

### 3.2 Effects of landscape‐scale land use on nesting females and reproductive output

We found no significant effects on the number of nesting females, nor any of the measures of offspring numbers, of either landscape type (conventional, organic farming, or pasture rich), the length of field borders, or the area of OSR field within 500 m of nests (all results *p *>* *.10).

### 3.3 Effects of local resource availability on nesting females and reproductive output

**Figure 4:**
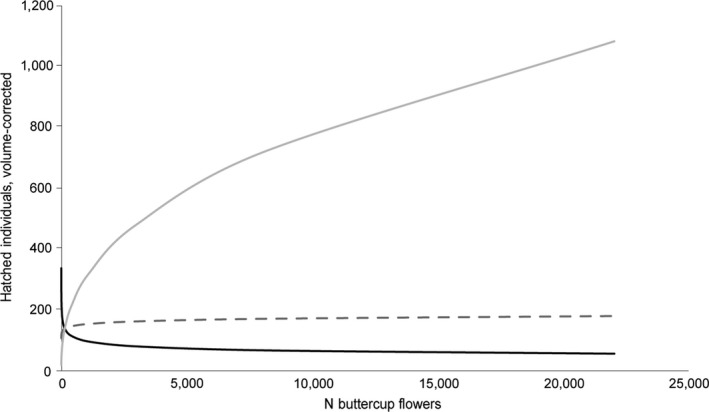
Model predicted number of offspring per sector, adjusted for the larger size of female pupae, in relation to the number of oak trees (*Quercus robur*) and flowering buttercups (*Ranunculus* spp.) within 100 m from *Osmia bicornis* nests. Oak and buttercup interacted significantly to explain the number of offspring per sector. Black line show the effect of buttercup at the minimum abundance of oak counted during field surveys (*N* = 0), dashed line at the mean abundance (*N* = 1.6), and gray line at the maximum abundance of oak (*N* = 10). For results of statistical analyses, see Table 2

We found that the amount of two highly preferred flower resources oak and buttercup interacted to explain the number of offspring per sector, when adjusted for the larger volume of female cells by a factor of 1.6 (see Section 2; Table 2). The interaction shows that abundance of buttercup had a positive effect on the number of offspring only when the number of oaks was approximately above the mean for the surveyed sectors (Figure 4). We found nonsignificant trends for the same interaction to affect the number of nesting females per sector, the total number of provisioned brood cells, the number of hatched offspring, and the number of female offspring (Table 2).

**Table 2:** Results for the fixed effects on response variables related to several aspects of *O. bicornis* nesting population and reproduction, modeled at the sector level

Aspect to be analyzed	Response variable	*N*	Random structure	Distribution	Offset variable	Log oak	Log other trees	Log buttercup	Log Brassicaceae	Log oak × log buttercup
Nesting population	*N* nesting females	17	Olrf	Poisson	−	EC = 0.17 ± 0.38 *F*(1,12) = 0.84 *p* = .38	EC = −0.055 ± 0.13 *F*(1,12) = 0.18 *p* = .68	EC = −0.032 ± 0.063 *F*(1,12) = 0.26 *p* = .62	EC = 0.029 ± 0.037 *F*(1,12) = 0.61 *p* = .45	EC = 0.21 ± 0.10 *F*(1,11) = 4.31 *p* = .062
Reproduction of population	Log *N* cells built	17	−	Normal	−	EC = 0.13 ± 0.19 *F*(1,12) = 0.43 *p* = .52	EC = −0.087 ± 0.14 *F*(1,12) = 0.38 *p* = .55	EC = −0.028 ± 0.067 *F*(1,12) = 0.17 *p* = .68	EC = 0.020 ± 0.039 *F*(1,12) = 0.26 *p* = .62	EC = 0.21 ± 0.11 *F*(1,11) = 3.67 *p* = .082
Reproduction of population	Log *N* hatched offspring	17	−	Normal	−	EC = 0.16 ± 0.21 *F*(1,12) = 0.84 *p* = .38	EC = −0.094 ± 0.15 *F*(1,12) = 0.11 *p* = .74	EC = −0.042 ± 0.071 *F*(1,12) = 0.42 *p* = .53	EC = 0.023 ± 0.042 *F*(1,12) = 0.96 *p* = .35	EC = 0.23 ± 0.12 *F*(1,11) = 3.75 *p* = .079
Reproduction of population	Log (*N* male + 1.6 × N female offspring)	17	−	Normal	−	EC = −1.21 ± 0.65 *F*(1,11) = 3.48 *p* = .09	EC = −0.15 ± 0.13 *F*(1,11) = 1.31 *p* = .28	EC = −0.18 ± 0.090 *F*(1,11) = 3.97 *p* = .072	EC = 0.045 ± 0.037 *F*(1,11) = 1.48 *p* = .25	EC = 0.25 ± 0.11 *F*(1,11) = 4.78 *p* = **.051**
Reproduction of population	*N* female offspring	17	−	Normal	−	EC = 0.0057 ± 0.23 *F*(1,12) = 0.00 *p* = .98	EC = −0.016 ± 0.17 *F*(1,12) = 0.01 *p* = .93	EC = −0.0076 ± 0.080 *F*(1,12) = 0.01 *p* = .93	EC = 0.027 ± 0.047 *F*(1,12) = 0.32 *p* = .58	EC = 0.27 ± 0.13 *F*(1,11) = 4.42 *p* = .059
Sex ratio of offspring	*N* female/*N* hatched	17	Olrf	Binomial	−	EC = −0.19 ± 0.10 *F*(1,10.9) = 3.38 *p* = .093	EC = 0.11 ± 0.076 *F*(1,10.7) = 2.11 *p* = .18	EC = 0.051 ± 0.036 *F*(1,10.7) = 1.98 *p* = .19	EC = 0.0051 ± 0.022 *F*(1,11.3) = 0.06 *p* = .82	EC = 0.049 ± 0.49 *F*(1,10.4) = 0.52 *p* = .49
Reproduction per female	*N* cells	17	Olrf	Poisson	(Log) *N* nesting females	EC = 0.015 ± 0.080 *F*(1,11.6) = 0.03 *p* = .86	EC = 0.0033 ± 0.058 *F*(1,11.7) = 0.00 *p* = .96	EC = −0.012 ± 0.028 *F*(1,11.7) = 0.20 *p* = .66	EC = 0.0054 ± 0.016 *F*(1,12.0) = 0.11 *p* = .75	EC = −0.012 ± 0.052 *F*(1,10.9) = 0.05 *p* = .83

Significant results in bold. Nonsignificant interactions were removed, and models rerun to attain results for component factors. Degrees of freedom estimated using Kenward–Rogers method. Olrf = observation‐lever random factor. EC = model estimated coefficient ± standard error. See text for further information on models.

The measure of reproductive output per nesting female (built cells) was not related to either abundance of oak or buttercup or their interaction. We found no significant effect of any of the flower resources on the proportion of female offspring per sector (Table 2). Neither Brassicaceae flowers, nor flowering trees other than oak, had any significant effect on any of the above models (Table 2).

### 3.4 Interacting effects of oak and season on foraging trip times and speed of nest‐building

In total, 160 measurements were included in the analyses of foraging trip times, each measurement representing the mean value of all trips recorded by one female bee at one specific day. The mean duration (±std) of trip times was 577.2 (±274.1) s. We found that effects on pollen foraging trip times of both abundance of oaks and that of buttercups changed with the season. More oaks lead to longer time spent foraging, and as the season progressed and oak trees ceased to flower, this effect was enhanced (positive effect of oak and of the interaction between the number of oaks and days, Table 3). More buttercups surrounding a nest led to shorter trips, and those trips grew even shorter as the season progressed and more plants went into flower (negative effect of buttercup and of the interaction between buttercup and day, Table 3). The additional model with predictors centered around the group‐level mean and the difference from the sector mean at nest pole level confirmed the results for the interaction between buttercup and day (posterior mean (PM) = −0.11; 95% CI = −0.22, −0.018; *P *=* *.032), while the effect of the interaction between oak and day showed a nonsignificant positive trend (PM = 0.11; 95% CI = −0.020, 0.22; *P *=* *.090).

**Table 3:** Results for the fixed effects on nest‐building and foraging trip times, showing posterior mean (PM), 95% lower and upper confidence intervals (CI), and *p*‐value

Response variable	*N*	Model	Dist.	Random structure	Factors at sector level							Factors at day or nest level			
					Log oak	Log other trees	Log buttercup	Log Brassicaceae	Day number	Log oak × day number	Log buttercup × day number	Log time between measurements	Log temp	Log time of day	Log rain
Log nest volume	259	MCMCglmm	Norm.	sector, nest pole (sector), nest (nest pole (sector))	PM = 0.018; CI = −0.16, 0.20; *p* = .87	PM = −0.079; CI = −0.25, 0.070; *p* = .34	PM = 0.0040; CI = −0.18, 0.17; *p* = .96	PM = 0.10; CI = −0.064, 0.26; *p* = .23	PM = −0.34; CI = −0.48, −0.20; *p* = <.001	PM = −0.13; CI = −0.23, −0.049; ***p*** = **.0040**	PM = −0.0020; CI = −0.11, 0.12; *p* = .97	PM = 0.21; CI = 0.12, 0.31; ***p*** = **<.001**	PM = 0.42; CI = 0.31, 0.56; ***p*** = **<.001**	NA	PM = −0.011; CI = −0.11, 0.087; *p* = .84
Log pollen foraging trip time	160	MCMCglmm	Norm.	sector, nest pole (sector), nest (nest pole (sector))	PM = 0.024; CI = −0.068, 0.12; *p* = .60	PM = 0.052; CI = −0.029, 0.14; *p* = .20	PM = −0.16; CI = −0.24, −0.069; *p* = .0040	PM = −0.043; CI = −0.13, 0.040; *p* = .29	PM = 0.16; CI = 0.098, 0.22; *p* = <.001	PM = 0.098; CI = 0.027, 0.8; ***p*** = **.010**	PM = −0.067; CI = −0.13, 0.0016; ***p*** = **.044**	NA	PM = −0.12; CI = −0.18, −0.063; ***p*** = **<.001**	PM = 0.10; CI = 0.036, 0.16; ***p*** = **.0020**	NA

All explanatory variables were standardized. Significant results in bold. Nest volume is used as a measure of nest‐building speed, with duration of nest‐building (time between measurements) included as a factor in the model. Nonsignificant interactions were removed, and models rerun to attain results for component factors. Regional temperature data were used in the model of nest‐building, and local temperature taken at each measurement was used in the model of trip times. NA = factor not applicable to model. See text for further information on models.

**Figure 5:**
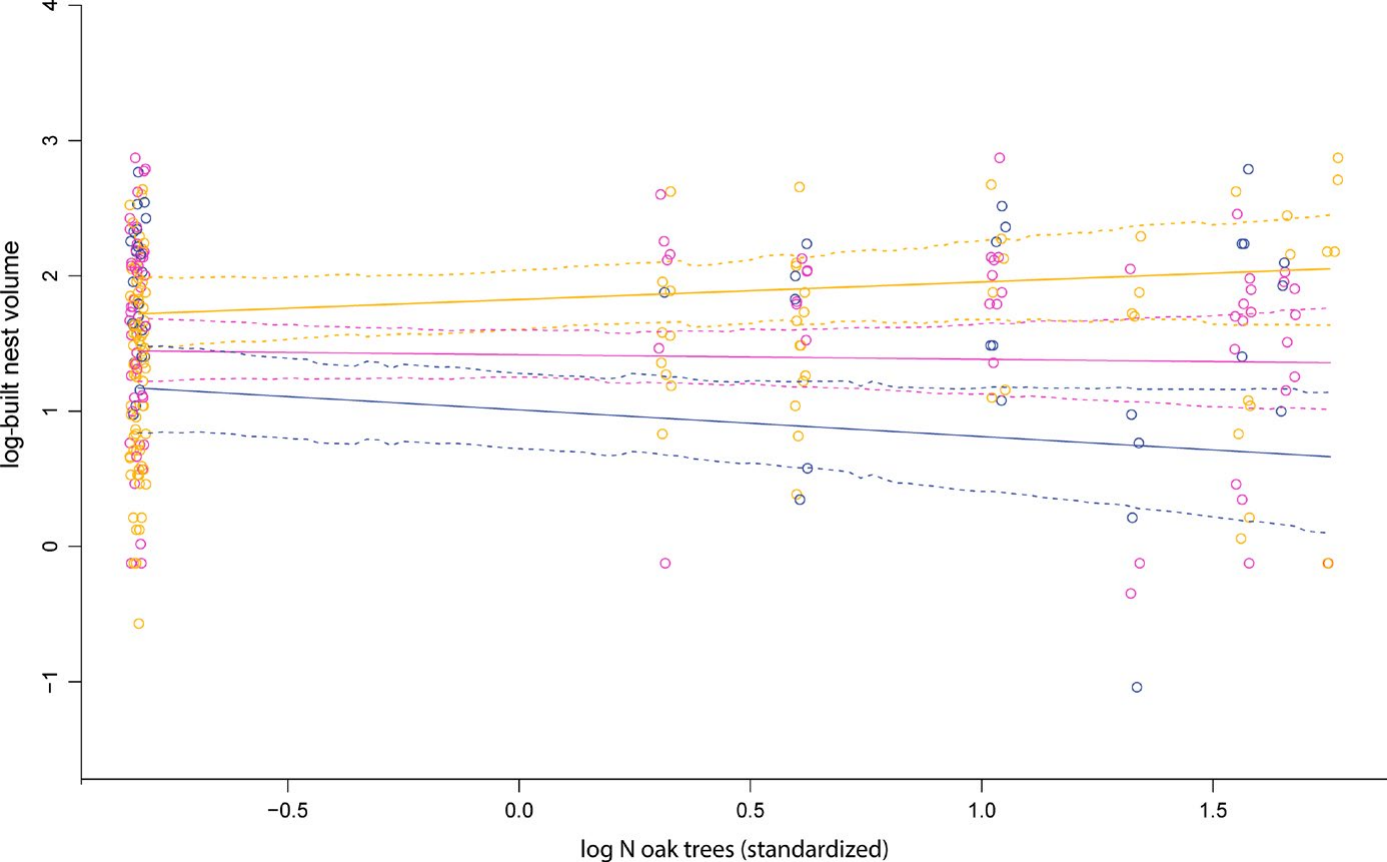
Nest volume (data points (circles) and model‐predicted volume (lines)) as a function of the number of oak trees surrounding nests at three points in time during the study period. Orange: early season 15 May, pink: mid‐season 25 May, blue: late season 4 June. Dashed lines show 95% CI. For results of statistical analyses, see Table 3

The analyses of nest‐building included 259 individual measurements, with a mean of 2.1 (±1.4) cm^3^ built per 24 hr, corresponding to 0.9 (±0.6) brood cells per 24 hr. The number of oak trees had a positive effect on the nest volume built by *O. bicornis* females, but the interaction with date showed that this effect tapered off as the season progressed (Table 3, Figure 5). The additional final model confirmed the result (negative effect of the interaction between mean oak per sector and day: PM = −0.13; 95% CI = −0.24, −0.036, *p *=* *.012). The result is associated with some uncertainty regarding the effect of the mean number of oaks in the landscape sector vs. at each nest pole, because for the full model including buttercup, the average number of oaks in the landscape sector no longer significantly interacted with day (PM = −0.097; 95% CI = −0.23, 0.033, *p *=* *.15). Instead, building speed tended to decrease over the season in response to the difference in the number of oaks per nest pole from the sector average (PM = −0.084; 95% CI = −0.17, 0.0018, *p *=* *.074). Similarly, there was a tendency for building speed to increase over the season in response to difference from the sector mean in abundance of buttercup at the nest pole (PM = 0.085; 95% CI = −0.00044, 0.17; *p *=* *.060); that is, very local deviations in resource availability may affect foraging behavior, also in otherwise resource‐rich sectors.

More OSR within 500 m of the nests led to faster nest‐building (positive interaction ORS and built nest volume: PM: 0.11; 95% CI: 0.011, 0.21; *p = *.044), and the result was confirmed in the additional model (PM = 0.11; 95% CI = 0.013, 0.22; *p *=* *.030). No other foraging resources, measured at local or landscape scales, showed any significant effect on trip times or speed of nest‐building.

## 4 DISCUSSION

The large‐scale declines of wild bees during past decades have led to an urgent need for evidence‐based conservation of habitat to aid bee population and communities (Potts et al., 2016). Bee habitats are often situated in farmland landscapes, where a range of interventions exist to promote bees and other insect pollinators. To properly design such interventions, it is necessary to understand how bee species use foraging resources. The present study improves the understanding of how bee foraging and fitness are affected by availability of temporally complementary resources. Although the species investigated, *Osmia bicornis*, is polylectic and can forage from several plant genera families, it largely relied on two plant species for pollen: one tree species (oak) and one herbaceous species (buttercup). We can thereby highlight the risks of conservation interventions (such as sown flower strips) which equate pollinator resource plants with “any flower” and provide only herbaceous plants (Wood et al., 2015). This is especially true for conservation of specialist bee species, where key plant species may not be exchangeable without negatively affecting reproduction due to metabolic constraints (Praz, Müller, & Dorn, 2008; Sedivy, Müller, & Dorn, 2011). Species with a short nesting season may instead be temporally restricted to a few plant species. Other studies have demonstrated resource complementation between crops and resource‐rich land cover types in the surrounding landscape, for example, crops and seminatural grasslands (Holzschuh et al., 2013; Rundlöf, Persson, Smith, & Bommarco, 2014), fallows, crops, and old fields (Mandelik et al., 2012), and organically managed farmland and native vegetation (Williams & Kremen, 2007). Here, we reveal that such resource complementation can be detected at smaller spatial scales (≤100 m) than the estimated foraging distance (Gathmann & Tscharntke, 2002). We also show that resources in seminatural grasslands in the wider landscape could not replace the two preferred pollen sources, thus stressing a need for management of seminatural habitats (and other bee habitats) to take on a species‐specific approach to better benefit a larger part of wild bee communities.

### 4.1 Pollen provisioning and resource availability

A large part of the species found in pollen samples were trees and shrubs. Apart from the clear preference for oak and buttercup, we found smaller amount of pollen from, for example, *Acer* spp., *Aesculus castanum*, lignose *Rosaceae*,* Sinapis/Brassica* spp., and *Salix*. The latter may have been used as nectar sources (Jauker et al., 2012; Radmacher & Strohm, 2010; Raw, 1974). Our results thus confirm the general importance of lignose vegetation for solitary bees in farmland landscapes. The use of trees and shrubs has likely been underestimated based on previous observations of flower visits, compared to the data on actual collected pollen (Wood et al., 2016). Our results also confirm that despite being polylectic, pollen provisions of *O. bicornis* and some other *Osmia* species are generally of low species diversity (MacIvor, Cabral, & Packer, 2014; Radmacher & Strohm, 2010; Raw, 1974).

The preference for oak and buttercup pollen could be caused by their nutritional value and digestive adaptations to their chemical composition (Roulston & Cane, 2000; Sedivy et al., 2011) and/or adaptations of the pollen‐carrying structures to pollen size and surface structure (Thorp, 1979, 2000). Pollen grains of oak and buttercup are very similar in size, shape, and surface (Punt & Clark, 1984; Reille, 1992), which could indicate that *O. bicornis* is adapted to efficiently collect and transport pollen of this morphology.

### 4.2 Effects of local resource availability and landscape type on nesting females and reproduction

We found that the local abundance of buttercup had a positive effect on the number of offspring (adjusted for the larger size of female offspring) only when the number of oaks in a sector was above the mean, in this case 1.6 oaks. We found nonsignificant trends for the same result for several other measures of *O. bicornis* reproduction. Together with results from pollen analyses, this suggests that these two resources are indeed complementary. In sites with oaks, buttercup can thus help cater for the nesting *O. bicornis* population once oak flowering declines. Potentially, *O. bicorni*s select nesting sites based on preferred early flower resources (in this case oak), and later flowering resources are needed to sustain a high reproduction of the nesting population.

The effects found appear despite the method of “seeding” the nests with *O. bicornis* pupae. The number of nesting females ranged from three to 17, which means that in the poorer sectors, some of the eight transplanted females likely left to nest elsewhere (or died), while “wild” individuals were attracted to the trap nests at richer sectors. Thus, we might have detected stronger effects of flower resources on the nesting population and offspring if we had not seeded the nests. Effects of local resource availability on the number of nest‐building females and reproduction per female have previously been reported for *O. lignaria*, using the number of established nests as a proxy for the number of nesting females (Williams & Kremen, 2007). Here, we used the number of simultaneously recorded nest‐building females, that is, a more conservative measure.

Contrary to expectations, we did not find any effects on the number of nesting females or offspring of either farming regime (conventional vs. organic farming or pasture‐rich landscape sectors) or availability of potentially resource‐rich habitats (field borders, OSR) within 500 m. This could imply that *O. bicornis* foraging range is shorter than previous studies suggest (Gathmann & Tscharntke, 2002) and/or that our landscape measures of land use do not capture variation in the few species mainly used for foraging. In both cases, the land‐use descriptors may be poor indicators of the amount of flower resources available to bees. Interestingly, other authors (Dainese et al., 2018; Holzschuh et al., 2013) have reported on pollen foraging and positive effects of OSR on population size of *O. bicornis*. We did not find any effect of OSR on the nesting population and offspring. One reason for this apparent discrepancy may be that *Osmia* populations in other regions (Germany and The Netherlands) have stronger preference for Brassicaceae pollen or that alternative resources were lacking during OSR flowering. In our study region, OSR flowering coincided with oak and buttercup. Another possibility is that OSR is used mainly for nectar foraging in our region and that alternative nectar sources were abundant. We did, however, find that OSR within 500 m from nests had a positive effect on nest‐building speed (see below).

We expected sex ratio to be more skewed toward females when early‐season resource availability was higher. Such results have been documented for leafcutter bees *Megachile rotundata* and *M. apicalis* (Kim, 1999; Peterson & Roitberg, 2006). Similar to leafcutter bees, *Osmia* lay female eggs before male ones (Giejdasz et al., 2016; Raw, 1972) and female larvae require a larger pollen provision for development compared to males (Radmacher & Strohm, 2010; Seidelmann, 2014). A large production of female offspring would therefore benefit from timing of the bee's phenology to abundant pollen resources in early season. In this study, early nesting was well matched in time to pollen release from oak, offering a flush of superabundant pollen. Oak provides substantially more pollen than, for example, buttercup (Broström et al., 2008; Mazier et al., 2012). However, we did not find any effects of oak on sex ratio. The reason may be that the period of laying female eggs extends beyond flowering of oak. *Osmia bicornis* nests can indeed be initiated with female eggs throughout the nesting season, although the proportion of females per nest has been shown to decrease with time (Giejdasz et al., 2016). We found a nonsignificant trend for the number of female offspring per site to be positively affected by the interaction of oak with buttercup; that is, female offspring showed the same response to flower resources as did the total number of offspring. This could indicate that oak and buttercup are equally good when catering for female offspring and are used complementary. Both female and male offspring numbers may also have been affected by the abundance of flower resources before nesting, because newly hatched females rely on pollen to, for example, increase lipid content to aid egg maturation (Cane, 2016). Thus, abundance of early spring pollen sources, for example, *Salix* spp., could have affected sex ratio, fitness of females, and choice of nesting site, without affecting pollen provisioning for offspring.

### 4.3 Effects of local resource availability on foraging trip times and nest‐building changed with season

We found that more oaks lead to longer time spent foraging and that foraging trips grew even longer in sectors with more oaks as the season progressed and oak flowering declined. The opposite was found for buttercup. We also found that more oaks led to faster nest‐building early in the season and that this effect tapered off through the nesting season. Although in general we would expect shorter foraging trips when resources are more abundant (Pope & Jha, 2018; Redhead et al., 2016), we suggest that in accordance with central‐place foraging theory (Olsson & Bolin, 2014; Olsson et al., 2015), the superabundant but spatially separated resource supplied by oaks prompts bees to fly and stay longer in patches in order to return with a heavier pollen load. This could also explain why nest‐building speeds were higher at the same time as foraging times were longer in response to oak. The increasingly positive effect of oaks on foraging times through the season could indicate that *O. bicornis* females in this region are reluctant to switch to alternative resources, even when possibly only distant oaks provide pollen and/or pollen becomes more time‐consuming to collect. Buttercup, a more evenly scattered resource, instead show the expected relation; more buttercup leading to shorter foraging trips. As mentioned above, the physiological status of nesting females partly depends on pollen intake prenesting (Cane, 2016; O'Neill et al., 2015). This may possibly obscure other effects, especially at the end of the nesting season when females are worn out.

The positive effect of the area of OSR in the landscape on nest‐building, in combination with the low abundance of Brassicaceae pollen in nest provisions and lack of effect on pollen foraging trip times, indicates that OSR was mainly used for nectar foraging. Similarly, Jauker et al. (2012) found OSR to benefit offspring production in spite of not being abundantly used in nest provisions. Hence, OSR can be an important complementary resource for *Osmia* in farmland landscapes even if not used for pollen provisioning.

Effects of local resource availability and farming intensity on speed of nest‐building have previously been reported for two oligolectic species, *Hoplitis adunca* and *Chelostoma rapunculi* (Zurbuchen, Cheesman et al., 2010), and one polylectic species, *O. lignaria*, (Williams & Kremen, 2007). Williams and Kremen (2007) also found that organic farming could buffer against the negative effect of distance to preferred native pollen plants, because bees switched to alternative pollen sources found at organic farms. We could not test this interaction, because abundance of oak was significantly related to landscape type.

### 4.4 Conclusions and implications for bee conservation in farmland landscapes

Local (≤100 m) variation in two preferred pollen resources (oak and buttercup) affected the speed of nest provisioning and a measure of total amount of offspring, thereby potentially population persistence of *O. bicornis*, despite alternative resource‐rich habitats being available at larger spatial scales. All study sectors were situated within a region of relatively small‐scale mixed farming, with linear noncrop elements and small woodlots. It is therefore interesting that local resource distribution had detectable effects on a polylectic species and was not overrun by the availability of resources in the wider landscape. We expect that effects of the immediate foraging landscape could be even stronger in landscapes with more intensive farming practices and less structural complexity, because of a decreased likelihood of complementary foraging resources found in such landscapes compared to those studied here (cf Scheper et al., 2013).

Our study highlights the benefit of maintaining landscape heterogeneity in the form of permanent field borders and grasslands containing woody vegetation, here particularly oak, that provide pollen resources. Hence, some agri‐environment schemes earlier implemented in Sweden that resulted in the removal of woody vegetation may rather have been detrimental for biodiversity (Riksantikvarieämbetet, 2017). In contrast, retaining or increasing the number of trees and shrubs, in particular in otherwise impoverished agricultural landscapes, may boost populations of wild pollinators and potentially enhance the ecosystem service they provide. Our results also point to the importance of tailoring flower strips and similar interventions to aid pollinators and other farmland wildlife (Wood et al., 2015, 2016), both to groups of species and to landscapes, depending on existing resources and land use. For example, including buttercup would highly increase the nutritional value of flower strips for *O. bicornis* in combination with existing oak trees or schemes to regenerate oak populations, and OSR may provide an important resource in combination with seminatural habitats (Holzschuh et al., 2013; Jauker et al., 2012). Effect of conservation actions on pollinator communities will thus depend on the specific content of created habitats, availability of other essential or temporally complementary resources, as well as the spatial scale of implementation.

## CONFLICT OF INTEREST

None declared.

## AUTHORS’ CONTRIBUTIONS

ASP and HGS planned and designed the study; ASP collected field data; ASP and FM collected pollen data; ASP and HGS analyzed data; ASP led the writing of the manuscript. All authors contributed to the drafts and gave the final approval for publication.

## DATA ACCESSIBILITY

Data used for analyses in this study will be archived at Dryad Digital Repository: https://doi.org/10.5061/dryad.dj454vt.
