# Supplementary material for: When beggars are choosers—How nesting of a solitary bee is affected by temporal dynamics of pollen plants in the landscape
Source: Ecol Evol. 2018 May 15;8(11):5777–91. doi: 10.1002/ece3.4116 (PMC6010912; doi:10.1002/ece3.4116)
Supplement: Supplementary file 1 [file ECE3-8-5777-s001.docx]

**Electronic Appendix**

Results from correlations between land cover variables during 2008 at 100 m, 500 m and 1000 m radius in the 18 landscape sectors studied. A. Arable includes annual crops, leys and fallow. B. Pasture is permanent grazed pasture: C. Organic fields include organically managed crop fields and lays. D. Oilseed rape is in these landscape sectors winter sown oilseed rape. Data was obtained from the Integrated Administration and Control System (IACS, Swedish Board of Agriculture).

A.

| Land cover | Arable 500 | Arable 1000 |
| --- | --- | --- |
| Arable 100 | r=0.73, P<0.0001 | r=0.65, P<0.0001 |
| Arable 500 |  | r=0.65, P<0.0001 |

B.

| Land cover | Pasture 500 | Pasture 1000 |
| --- | --- | --- |
| Pasture 100 | r=0.84, P<0.0001 | r=0.84, P<0.0001 |
| Pasture 500 |  | r=0.96, P<0.0001 |

C.

| Land cover | Organic 500 | Organic 1000 |
| --- | --- | --- |
| Organic 100 | r=0.94, P<0.0001 | r=0.83, P<0.0001 |
| Organic 500 |  | r=0.91, P<0.0001 |

D.

| Land cover | Oilseed rape 500 | Oilseed rape 1000 |
| --- | --- | --- |
| Oilseed rape 100 | r=0.79, P<0.0001 | r=0.65, P=0.0038 |
| Oilseed rape 500 |  | r=0.80, P<0.0001 |
